# Maternal mortality in six low and lower-middle income countries from 2010 to 2018: risk factors and trends

**DOI:** 10.1186/s12978-020-00990-z

**Published:** 2020-12-17

**Authors:** Melissa Bauserman, Vanessa R. Thorsten, Tracy L. Nolen, Jackie Patterson, Adrien Lokangaka, Antoinette Tshefu, Archana B. Patel, Patricia L. Hibberd, Ana L. Garces, Lester Figueroa, Nancy F. Krebs, Fabian Esamai, Paul Nyongesa, Edward A. Liechty, Waldemar A. Carlo, Elwyn Chomba, Shivaprasad S. Goudar, Avinash Kavi, Richard J. Derman, Sarah Saleem, Saleem Jessani, Sk Masum Billah, Marion Koso-Thomas, Elizabeth M. McClure, Robert L. Goldenberg, Carl Bose

**Affiliations:** 1 Department of Pediatrics, University of North Carolina School of Medicine, 101 Manning Drive, CB 7596, Chapel Hill, NC 27599-7596 USA; 2 RTI International, Durham, NC USA; 3 Kinshasa School of Public Health, Kinshasa, Democratic Republic of Congo; 4 Lata Medical Research Foundation, Nagpur, India; 5 Adjunct Faculty Medical Research, Datta Meghe Institute of Medical Sciences, Sawangi, India; 6 Boston University School of Public Health, Boston, MA USA; 7 Instituto de Nutrición de Centroamérica y Panamá, Guatemala City, Guatemala; 8 University of Colorado School of Medicine, Denver, CO USA; 9 Moi University School of Medicine, Eldoret, Kenya; 10 Indiana School of Medicine, University of Indiana, Indianapolis, IN USA; 11 University of Alabama at Birmingham, Birmingham, AL USA; 12 University Teaching Hospital, Lusaka, Zambia; 13 KLE Academy Higher Education and Research, J N Medical College Belagavi, Belgaum, Karnataka India; 14 Thomas Jefferson University, Philadelphia, USA; 15 Aga Khan University, Karachi, Pakistan; 16 Maternal and Child Health Division (icddr, b), Dhaka, Bangladesh; 17 Sydney School of Public Health, The University of Sydney, Sydney, NSW 2006 Australia; 18 Eunice Kennedy Shriver National Institute of Child Health and Human Development, Bethesda, MD USA; 19 Department of Obstetrics and Gynecology, Columbia University School of Medicine, New York, NY USA

**Keywords:** Low-resource countries, Maternal mortality, Sustainable development goals, Global network

## Abstract

**Background:**

Maternal mortality is a public health problem that disproportionately affects low and lower-middle income countries (LMICs). Appropriate data sources are lacking to effectively track maternal mortality and monitor changes in this health indicator over time.

**Methods:**

We analyzed data from women enrolled in the NICHD Global Network for Women’s and Children’s Health Research Maternal Newborn Health Registry (MNHR) from 2010 through 2018. Women delivering within research sites in the Democratic Republic of Congo, Guatemala, India (Nagpur and Belagavi), Kenya, Pakistan, and Zambia are included. We evaluated maternal and delivery characteristics using log-binomial models and multivariable models to obtain relative risk estimates for mortality. We used running averages to track maternal mortality ratio (MMR, maternal deaths per 100,000 live births) over time.

**Results:**

We evaluated 571,321 pregnancies and 842 maternal deaths. We observed an MMR of 157 / 100,000 live births (95% CI 147, 167) across all sites, with a range of MMRs from 97 (76, 118) in the Guatemala site to 327 (293, 361) in the Pakistan site. When adjusted for maternal risk factors, risks of maternal mortality were higher with maternal age > 35 (RR 1.43 (1.06, 1.92)), no maternal education (RR 3.40 (2.08, 5.55)), lower education (RR 2.46 (1.54, 3.94)), nulliparity (RR 1.24 (1.01, 1.52)) and parity > 2 (RR 1.48 (1.15, 1.89)). Increased risk of maternal mortality was also associated with occurrence of obstructed labor (RR 1.58 (1.14, 2.19)), severe antepartum hemorrhage (RR 2.59 (1.83, 3.66)) and hypertensive disorders (RR 6.87 (5.05, 9.34)). Before and after adjusting for other characteristics, physician attendance at delivery, delivery in hospital and Caesarean delivery were associated with increased risk. We observed variable changes over time in the MMR within sites.

**Conclusions:**

The MNHR is a useful tool for tracking MMRs in these LMICs. We identified maternal and delivery characteristics associated with increased risk of death, some might be confounded by indication. Despite declines in MMR in some sites, all sites had an MMR higher than the Sustainable Development Goals target of below 70 per 100,000 live births by 2030.

**Trial registration:**

The MNHR is registered at NCT01073475.

## Plain English summary

Reducing maternal mortality is a global health priority. Maternal mortality disproportionately affects women in low and lower-middle income countries, and many of these deaths are preventable. We describe maternal death in research sites in the Democratic Republic of Congo, Guatemala, India (Nagpur and Belagavi), Kenya, Pakistan, and Zambia. We emphasize that the number of women dying in these countries is higher than the targets set out in the World Health Organization’s Sustainable Development Goals for 2030. We describe large differences between countries in terms of the ratio of maternal death. We identify maternal and delivery characteristics associated with increased risk of death, although some of the characteristics might be influenced by other factors.

## Background

Reducing maternal mortality is a global health priority. The Sustainable Development Goals (SDGs) aim for a reduction of the maternal mortality ratio (MMR) to below 70 per 100,000 live births by 2030 [1]. If the SDGs are met by 2030, the lives of an estimated 1.6 million mothers will be saved [2]. Maternal mortality is not only a health indicator, but also a key indicator of country development because maternal deaths have an important social and economic impact [3–5]. Maternal mortality disproportionately affects women in low and lower-middle income countries (LMICs) where most of the maternal deaths are from preventable causes [6].

Despite the majority of maternal deaths occurring in LMICs (MMR of 479 for low income countries compared to 41 in high income countries), robust systems for data collection and health indicator tracking are lacking [7]. This makes reliable tracking of MMRs difficult, despite global attention to the problem [8]. Also, controversy still exists regarding the optimal way to monitor maternal mortality [9]. In areas where health registries are lacking, the MMR can be estimated through a series of modelling strategies which rely on local data sources [8, 10, 11]. When data are sparse, such as in LMICs, these strategies rely on complex models with several poorly defined variables and weakly justified assumptions that lead to low precision in the final results [10, 12]. Therefore, primary datasets that reliably track the MMR in LMICs are urgently needed to provide a more robust evidence base for evaluating and tracking maternal mortality [13].

In this manuscript, we describe maternal mortality in 6 LMICs from a defined geographic, community-based, prospectively collected maternal health registry that captures data on all women delivering within or outside of facilities. This longitudinal dataset describes maternal deaths over a 9-year period. We examine maternal characteristics associated with maternal deaths, causes of maternal death and evaluate site specific trends in the MMR over time.

## Methods

We analyzed data from the *Eunice Kennedy Shriver* National Institute of Child Health and Human Development (NICHD) Global Network (GN) for Women’s and Children’s Health Research Maternal Newborn Health Registry (MNHR) [14]. The MNHR is a multi-country pregnancy registry including research sites in the Democratic Republic of Congo (DRC; North and South Ubangi Provinces); Guatemala (Western Highlands); India (Belagavi and Nagpur); Kenya (Western region); Pakistan (near the city of Karachi); and Zambia (south and east of the capital city of Lusaka). The study population includes both peri-urban and rural settings.

We included all pregnancies from January 2010 to December 2018, expanding on previously published results from 2010 to 2013 [15]. MNHR data, were collected from pregnant women who reside in or deliver within study clusters through various methods, including detailed interviews conducted by trained study staff, as well as abstraction from medical records. Women were identified for inclusion as early as possible during their pregnancy, then screened and consented. We collected baseline maternal characteristics at the time of entry into the registry. Additional antenatal and delivery characteristics were recorded within 3–7 days of delivery and postpartum details were collected at home or clinic visits 6 weeks after delivery. We excluded women who were lost to follow -up prior to delivery or those with missing data on maternal status at 42 days after the end of the pregnancy.

We defined maternal death in accordance with the World Health Organization definition of death of the mother while pregnant or within 42 days of the end of the pregnancy. We defined MMR as maternal deaths/100,000 live births. To calculate the MMR, we included all maternal deaths, regardless of the birth outcome (miscarriage, stillbirth, medical termination of pregnancy [MTP], live birth and unknown birth outcomes) in the numerator. The denominator is live born infants. The 95% confidence interval for the MMR is approximated using the variance of the proportion of maternal deaths for each site and year. We defined fetal malposition as transverse lie, oblique lie or breech presentation.

We explored the relationship between characteristics and overall mortality using log-binomial generalized linear models with generalized estimating equations to obtain point and interval estimates of risk ratios for mortality modeled as a function of each characteristic independently while controlling for the correlation within clusters. For the purpose of the models, we evaluated the outcome of maternal death vs. women who survived to 42 days after the end of pregnancy. We included women who experienced all birth outcomes (miscarriage, stillbirth, live birth and unknown birth outcomes) in the models. Women without the characteristic (e.g. labor not obstructed) served as the reference group.

Next, we ran a multivariable regression model to determine the maternal, pregnancy related, delivery and antepartum factors that were associated with maternal death. Medical and social variables collected at the time of enrollment or around the time of delivery that could be associated with maternal mortality and were reliably collected in the MNHR were included. We included: maternal age, maternal education, parity, antenatal care (ANC), birth attendant, delivery mode, obstructed labor, fetal malposition, hemorrhage and hypertensive disorders. We defined severe antepartum hemorrhage as blood loss greater than 1000 cm^3^ (cc) of blood prior to delivery. Factors with significant missing fields were excluded. We adjusted the model further for research site and accounted for correlation of outcomes within clusters. Data are presented as adjusted risk ratios and 95% confidence intervals. In 2013, the MNHR began assigning cause of maternal deaths by collecting data describing factors associated with deaths. We assigned a cause of death from these data using a standardized, hierarchical, algorithm [16].

The MNHR had some notable differences in its population over time. The DRC site entered the MNHR in 2014. Throughout the 9-year study period, there was expansion and contraction of clusters within each country to meet the research needs of the GN. In order to limit external forces that might alter the study population by inclusion of different clusters, we described the MMR over time using a subset of data. This subset included only GN clusters that were consistent throughout the 9-year period in the Guatemala, India (Nagpur and Belagavi), Kenya, Pakistan, and Zambia sites. For the DRC site, we included women who lived in clusters that remained in the registry from 2014 to 2018. Because of the small number of maternal deaths and the variation in the MMR from year to year, we evaluated the MMR in overlapping periods of 3 years. This approach permitted evaluation using a running average. Because data are not available from the DRC site throughout the entire period of analysis, we report the total MMR with data from the DRC site included and excluded.

The Data Coordinating Center at RTI International (Durham, NC) performed all analyses using SAS, Inc. (Version 9.4). Institutional Review Boards or research ethics committees and Ministries of Health at each site approved the collection of data included in the MNHR. Prior to the initiation of data collection, we used sensitization meetings to gain local approval of study procedures at the community level. Individual participants gave informed consent. The NICHD appointed a data monitoring committee to annually review the MNHR.

## Results

**Fig. 1:**
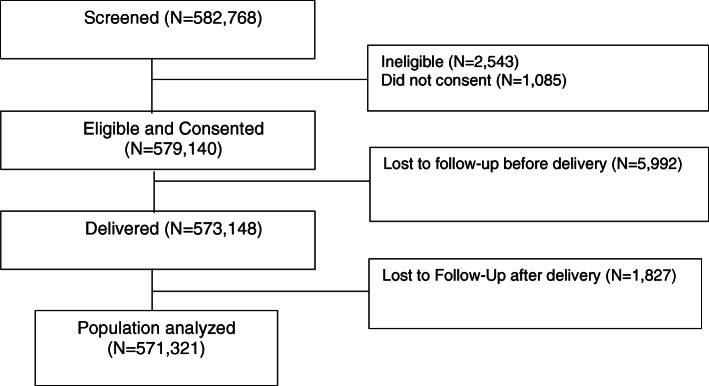
Enrollment Diagram

We screened 582,768 women for inclusion in the MNHR from 2010 to 2018. Of those screened, 579,140 (99.4%) were eligible and consented to be part of the MNHR, (Fig. 1). Of women who consented, 7819 (1.3%) were lost to follow up, leaving 571,321 (98.6%) for analysis. These pregnancies resulted in 576,685 outcomes (including multiple gestations): 19,080 (3%) miscarriages, 14,432 (3%) stillbirths, 7789 (1%) MTPs, 225 (< 0.1%) unknown birth outcomes and 535,159 (93%) live births (Table 1). We included data on 842 maternal deaths: 452 (53%) of the birth outcomes of the women who died were live births, 182 (21%) were stillbirths, 7 (< 1%) were miscarriages, 3 (< 1%) were MTPs, and 219 (25%) had unknown birth outcomes. Thus, of the 75% of cases where fetal or neonatal outcome were known, more than 20% were associated with stillbirth. We observed an MMR of 157 / 100,000 live births (95% CI 147, 167), with a range of MMRs from 97 (76, 118) in the Guatemala site to 327 (293, 361) in the Pakistan site.

**Table 1:** Overall pregnancy outcomes by site, 2010–2018, all clusters^a^

Characteristic	Overall, all sites	Democratic Republic of Congo	Guatemala	Belagavi	Nagpur	Kenya	Pakistan	Zambia
Pregnancies, n	571,321	31,896	83,320	135,384	87,395	73,904	96,760	62,662
Miscarriages, n	19,080	244	694	8280	3322	210	5877	453
Medical Termination of Pregnancy, n	7789	83	1	4362	1993	33	1303	14
Unknown birth outcomes, n	225	30	10	37	38	23	71	16
Stillbirths, n	14,432	1239	1522	3002	1775	1554	4164	1176
Live births, n	535,159	30,870	81,672	120,629	80,917	72,973	86,458	61,640
Maternal deaths, n	842	98	79	149	89	78	283	66
Total MMR(95% CI)^b^	157 (147, 167)	317 (257, 378)	97 (76, 118)	124 (106, 141)	110 (89, 131)	107 (83, 130)	327 (293, 361)	107 (82, 132)

^a^ Maternal Newborn Health Registry 2010–2018 deliveries, excluding women lost to follow-up prior to delivery or missing maternal status at 42 days

^b^ Maternal Mortality Ratio (MMR) is maternal deaths per 100,000 live births. The 95% confidence interval for the MMR is approximated using the variance of the proportion of maternal deaths for each site and year

We evaluated maternal characteristics associated with maternal death (Table 2). Women who were older than 35 years of age, those with no formal or only primary/secondary education compared to university or higher education, nulliparous or multiparous with > 2 pregnancies, compared to 1–2 prior pregnancies and those whose last pregnancy did not result in a live birth were at an increased risk for maternal death. Antenatal and delivery characteristics that were associated with an increased risk of maternal death included: limited or no ANC, hospital delivery, delivery by a physician, assisted vaginal or Caesarean delivery, obstructed labor, fetal malpresentation, severe antepartum hemorrhage (> 1000 cc blood loss), hypertensive disorders and intrapartum medical treatments such as antibiotics, corticosteroids, blood transfusions, removal of retained products, anticonvulsant medications, IV fluids, use of forceps/vacuum, and other surgeries or treatments (Table 3).

**Table 2:** Maternal characteristics by maternal status at 42 days after delivery, 2010–2018 all clusters

Characteristic^a^	N(%) by maternal status		Total	Risk Ratio(95% CI)^b^
	Women who died	Women alive six weeks after delivery		
Deliveries, N	842	570,479	571,321	
Maternal age	839	569,478	570,317	
< 20	65 (7.7)	71,758 (12.6)	71,823 (12.6)	0.7 (0.5, 0.9)
20–35	695 (82.8)	471,940 (82.9)	472,635 (82.9)	1.0
> 35	79 (9.4)	25,780 (4.5)	25,859 (4.5)	2.0 (1.6, 2.5)
Maternal education	841	568,765	569,606	
No formal education	360 (42.8)	137,325 (24.1)	137,685 (24.2)	3.8 (2.6, 5.6)
Primary/Secondary	458 (54.5)	391,879 (68.9)	392,337 (68.9)	1.9 (1.3, 2.8)
University +	23 (2.7)	39,561 (7.0)	39,584 (6.9)	1.0
Parity	836	566,711	567,547	
0	238 (28.5)	183,868 (32.4)	184,106 (32.4)	1.2 (1.0, 1.4)
1–2	273 (32.7)	240,049 (42.4)	240,322 (42.3)	1.0
> 2	325 (38.9)	142,794 (25.2)	143,119 (25.2)	1.7 (1.5, 2.1)
Last pregnancy did not result in a live birth	56/598 (9.4)	21,148/382,772 (5.5)	21,204/383,370 (5.5)	1.7 (1.3, 2.3)

^a^ The denominator used to determine the percentage of women with each characteristic varies due to missing data

^b^ Risk ratios were generated from models evaluating the outcome of women who died vs women who were alive six weeks after delivery. Generalized linear models were used to evaluate the relationship of potential factors and maternal mortality and to develop point and interval estimates of relative risk associated with these factors. Generalized estimating equations were used to account for the correlation of outcomes within cluster to develop appropriate confidence intervals. Unless otherwise noted, the reference group is women who did not have the listed characteristic

**Table 3:** Antenatal and delivery characteristics by maternal status at 42 days after delivery, 2010–2018 all clusters

Characteristic^a^	N(%) by maternal status		Total	Risk Ratio(95% CI)^b^
	Women who died	Women alive six weeks after delivery		
Deliveries, N	842	570,479	571,321	
No antenatal care (vs. any antenatal care)	62/834 (7.4)	20,426/570,105 (3.6)	20,488/570,939 (3.6)	1.6 (1.1, 2.4)
Fewer than 4 antenatal care visits (vs. > 4 visits)	339/605 (56.0)	201,105/447,076 (45.0)	201,444/447,681 (45.0)	1.3 (1.1, 1.7)
Birth attendant	620	570,351	570,971	
Physician	291 (46.9)	203,294 (35.6)	203,585 (35.7)	1.0
Nurse/Midwife/Health worker	131 (21.1)	203,325 (35.6)	203,456 (35.6)	0.4 (0.3, 0.5)
Traditional birth attendant	134 (21.6)	119,738 (21.0)	119,872 (21.0)	0.5 (0.4, 0.7)
Family/Self/Other	64 (10.3)	43,994 (7.7)	44,058 (7.7)	0.8 (0.6, 1.0)
Delivery location	623	570,402	571,025	
Hospital	335 (53.8)	244,926 (42.9)	245,261 (43.0)	1.0
Clinic/health center	113 (18.1)	170,980 (30.0)	171,093 (30.0)	0.4 (0.3, 0.5)
Home/other	175 (28.1)	154,496 (27.1)	154,671 (27.1)	0.6 (0.5, 0.8)
Delivery mode	612	545,759	546,371	
Vaginal	424 (69.3)	466,355 (85.5)	466,779 (85.4)	1.0
Vaginal assisted	19 (3.1)	5364 (1.0)	5383 (1.0)	3.3 (1.7, 6.5)
Caesarean section	169 (27.6)	74,040 (13.6)	74,209 (13.6)	2.7 (2.3, 3.3)
Obstructed labor	148/697 (21.2)	44,947/569,869 (7.9)	45,095/570,566 (7.9)	3.0 (2.3, 3.9)
Fetal Malpresentation	45/692 (6.5)	11,253/569,773 (2.0)	11,298/570,465 (2.0)	3.3 (2.4, 4.4)
Severe antepartum hemorrhage	71/695 (10.2)	8783/569,937 (1.5)	8854/570,632 (1.6)	6.7 (5.0, 9.0)
Hypertensive disorders	156/688 (22.7)	14,650/569,679 (2.6)	14,806/570,367 (2.6)	10.6 (8.5, 13.2)
Maternal treatment provided				
Maternal antibiotics	350/559 (62.6)	229,932/464,473 (49.5)	230,282/465,032 (49.5)	2.3 (1.8, 2.9)
Corticosteroids	43/439 (9.8)	10,225/413,905 (2.5)	10,268/414,344 (2.5)	4.0 (2.8, 5.9)
Oxytocics (including Misoprostol)	344/556 (61.9)	308,315/463,555 (66.5)	308,659/464,111 (66.5)	0.9 (0.7, 1.1)
Blood transfusion	186/560 (33.2)	9789/464,309 (2.1)	9975/464,869 (2.1)	20.9 (16.0, 27.2)
Removal of retained products	45/558 (8.1)	20,750/464,252 (4.5)	20,795/464,810 (4.5)	2.1 (1.4, 3.1)
Anticonvulsants/Magnesium sulfate	60/553 (10.8)	5692/464,277 (1.2)	5752/464,830 (1.2)	9.8 (7.2, 13.4)
V Fluids	182/258 (70.5)	90,672/173,017 (52.4)	90,854/173,275 (52.4)	2.4 (1.7, 3.4)
Forceps/vacuum	13/254 (5.1)	1908/172,354 (1.1)	1921/172,608 (1.1)	4.3 (2.4, 7.7)
Other surgery/treatment	22/255 (8.6)	9830/172,810 (5.7)	9852/173,065 (5.7)	1.7 (1.1, 2.7)

^a^ The denominator used to determine the percentage of women with each characteristic varies due to missing data

^b^ Risk ratios were generated from models evaluating the outcome of women who died vs women who were alive six weeks after delivery. Generalized linear models were used to evaluate the relationship of potential factors and maternal mortality and to develop point and interval estimates of relative risk associated with these factors. Generalized estimating equations were used to account for the correlation of outcomes within cluster to develop appropriate confidence intervals. Unless otherwise noted, the reference group is women who did not have the listed characteristic

We evaluated selected factors and their association with maternal death using a multivariable model (Table 4). We included site, maternal, antenatal and delivery characteristics in the model to determine the relationship with maternal mortality. After adjusting for differences in characteristics, we found the same direction and similar magnitude of association for age, education and parity. Obstructed labor, severe antepartum hemorrhage, and hypertensive disorders also still had an increased risk of death. However, when adjusted for other factors, malpresentation and ANC were no longer associated with risk of death. Of note, delivery location, birth attendance and delivery mode remained significant in the model. However, while physician delivery was still associated with an increased risk of maternal mortality compared to delivery by a nurse/midwife/health worker, it no longer had an increased observed risk compared to traditional birth attendants or family/self/other (RR confidence intervals include 1). Likewise, delivery in a hospital retained a significant association with increased maternal mortality compared to delivery in a clinic/health center, but not compared to home/other. Lastly, while caesarean delivery still showed an association with increased risk of maternal mortality compared to vaginal delivery, vaginal assisted delivery did not.

**Table 4:** Multivariable model of maternal status at 42 days after delivery controlling for site, maternal, antenatal and delivery characteristics, 2010–2018 all clusters

Characteristic	Overall p-value^a^	Risk Ratio (95% CI)^a^
Maternal age	0.0017	
< 20		0.64 (0.45, 0.89)
20–35		1.0
> 35		1.43 (1.06, 1.92)
Maternal education	<.0001	
No formal education		3.40 (2.08, 5.55)
Primary/Secondary		2.46 (1.54, 3.94)
University +		1.0
Parity	0.0031	
0		1.24 (1.01, 1.52)
1–2		1.0
> 2		1.48 (1.15, 1.89)
At least one antenatal care visit	0.1707	1.22 (0.92, 1.61)
Birth attendant	<.0001	
Physician		1.0
Nurse/Midwife/Health worker		0.61 (0.45, 0.84)
Traditional birth attendant		0.74 (0.50, 1.10)
Family/Self/Other		1.38 (0.91, 2.09)
Delivery location	<.0001	
Hospital		1.0
Clinic/health center		0.57 (0.44, 0.75)
Home/other		0.89 (0.62, 1.28)
Delivery mode	0.0048	
Vaginal		1.0
Vaginal assisted		1.58 (0.80, 3.12)
Caesarean section		1.60 (1.21, 2.13)
Obstructed labor	0.0062	1.58 (1.14, 2.19)
Fetal Malpresentation	0.1140	1.30 (0.94, 1.79)
Severe antepartum hemorrhage	<.0001	2.59 (1.83, 3.66)
Hypertensive disorders	<.0001	6.87 (5.05, 9.34)

^a^ A generalized linear model was used to evaluate the relationship of potential factors and maternal mortality and to develop point and interval estimates of relative risk associated with these factors after controlling for site and all other listed characteristics. Generalized estimating equations were used to account for the correlation of outcomes within cluster to develop appropriate confidence intervals. Unless otherwise noted, the reference group is women who did not have the listed characteristic

We identified hemorrhage (33%), infection (31%) and pre-eclampsia/eclampsia (16%) as the most common causes of maternal death overall in our population (Table 5). The primary causes of death varied by site and we observed wide site differences in the percentage of deaths attributable to these causes.

**Table 5:** Cause of maternal death by site, all clusters

Characteristic	Overall	DRC	Guatemala	Belagavi	Nagpur	Kenya	Pakistan	Zambia
Maternal deaths^a^, n	842	98	79	149	89	78	283	66
Maternal cause of death data available^b^, n (%)	436 (51.8)	97 (99.0)	46 (58.2)	50 (33.6)	43 (48.3)	35 (44.9)	139 (49.1)	26 (39.4)
Maternal cause of death, n (%)	436	97	46	50	43	35	139	26
Trauma	22 (5.0)	4 (4.1)	2 (4.3)	4 (8.0)	2 (4.7)	5 (14.3)	3 (2.2)	2 (7.7)
Abortion related	21 (4.8)	6 (6.2)	1 (2.2)	1 (2.0)	2 (4.7)	6 (17.1)	4 (2.9)	1 (3.8)
Preeclampsia/ Eclampsia	69 (15.8)	10 (10.3)	16 (34.8)	8 (16.0)	5 (11.6)	10 (28.6)	15 (10.8)	5 (19.2)
Hemorrhage	144 (33.0)	41 (42.3)	14 (30.4)	16 (32.0)	13 (30.2)	8 (22.9)	44 (31.7)	8 (30.8)
Infection	136 (31.2)	18 (18.6)	13 (28.3)	20 (40.0)	13 (30.2)	2 (5.7)	63 (45.3)	7 (26.9)
Medical condition coincident to pregnancy	23 (5.3)	9 (9.3)	0 (0.0)	1 (2.0)	4 (9.3)	1 (2.9)	6 (4.3)	2 (7.7)
Unknown	21 (4.8)	9 (9.3)	0 (0.0)	0 (0.0)	4 (9.3)	3 (8.6)	4 (2.9)	1 (3.8)

^a^ Maternal Newborn Health Registry (MNHR) 2010–2018 deliveries, excluding women lost to follow up prior to delivery or missing maternal status at 42 days

^b^ Maternal cause of death data collected from late 2013 to 2018. Cause of death determined by a standardized, heirarchical algorithm [16] in which one cause of death is identified, therefore, categories are mutually exclusive

**Fig. 2:**
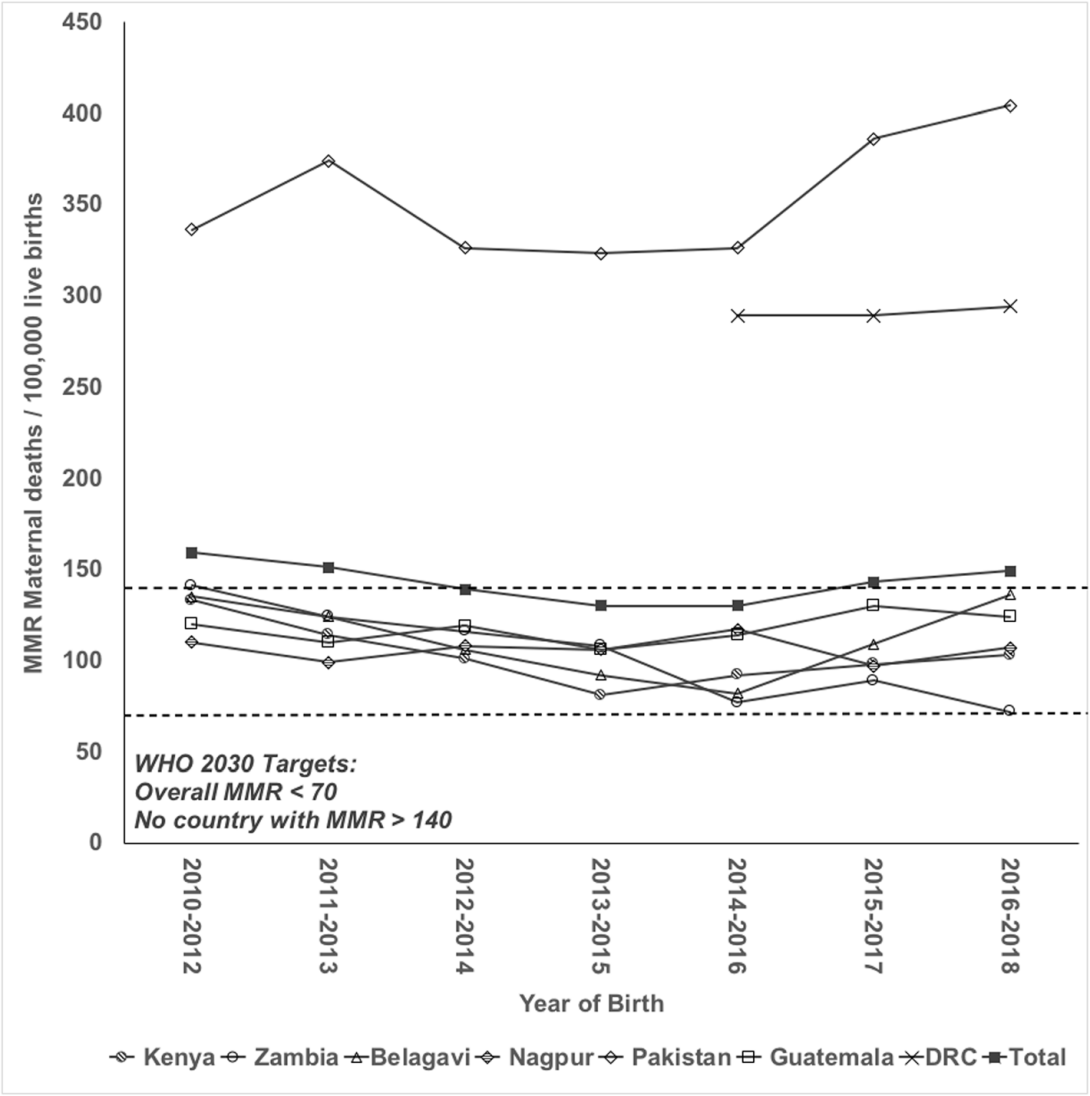
Running average of maternal mortality ratio^1^ by site and years, 2010–2018 ongoing clusters^2^. ^1^ Maternal Mortality Ratio (MMR) is maternal deaths per 100,000 live births. ^2^ Maternal Newborn Health Registry 2010–2018 deliveries excluding women lost to follow-up prior to delivery or missing maternal status at 42 days. Clusters collecting data during the entire period of 2010–2018, or in the DRC where clusters collecting data from 2014 to 2018 are represented. DRC is excluded from total, since data not known prior to 2014

Of the overall study population, we included 466,772 (81.7%) for analysis of trends in the MMR over time (Table 6). The total MMR in the ongoing clusters was 158 (147, 169). When we excluded the DRC site from the overall MMR trend, we observed variance in the MMR from 130 (112, 148) to 159 (139, 178). We observed site variation of MMR over time (Fig. 2). The Zambia site varied in the 3 year running averages from 141 (91, 192) in the earliest interval to 72 (36, 108) in the latest interval. The Kenya site varied from 133 (90, 176) in the earliest interval to 103 (61,144) in the latest interval. The Pakistan site 3 year running averages ranged from 336 (265, 408) to 404 (321, 488). The sites in Guatemala, Belagavi and Nagpur, India had similar MMR from the beginning to the end of the study period. Only 5 years of data were available for the DRC, and the MMR varied little from 289 (213, 365) to 294 (220, 329).

**Table 6:** Trend in maternal mortality ratio by site and combined years, 2010–2018 in ongoing clusters^a^

Characteristic	Overall, all sites	Overall, excluding DRC	DRC	Guatemala	Belagavi	Nagpur	Kenya	Pakistan	Zambia
Ongoing clusters^a^									
Births, n	466,772	434,876	31,896	60,434	88,684	84,241	73,904	64,951	62,662
Maternal deaths, n	694	596	98	69	95	84	78	204	66
Deliveries, n	471,272	438,806	32,466	60,832	89,300	84,872	74,793	65,710	63,299
Live births, n	438,855	407,985	30,870	59,198	78,265	77,968	72,973	57,941	61,640
Total MMR(95% CI)^a^	158 (147, 169)	146 (135, 157)	317 (257, 378)	117 (90, 143)	121 (100, 143)	108 (86, 129)	107 (83, 130)	352 (309, 395)	107 (82, 132)
MMR, maternal deaths per 100,000 live births (95% CI))^b^									
2010–2012	159 (139, 178)	159 (139, 178)		120 (68, 172)	135 (100, 170)	110 (74, 146)	133 (90, 176)	336 (265, 408)	141 (91, 192)
2011–2013	151 (132, 169)	151 (132, 169)		110 (63, 158)	124 (91, 158)	99 (64, 134)	114 (74, 154)	374 (299, 450)	124 (76, 172)
2012–2014	139 (121, 158)	139 (121, 158)		119 (71, 168)	106 (73, 139)	108 (71, 144)	101 (62, 141)	326 (255, 397)	116 (69, 163)
2013–2015	130 (112, 148)	130 (112, 148)		106 (63, 150)	92 (59, 125)	106 (70, 142)	81 (45, 117)	323 (253, 393)	108 (63, 153)
2014–2016	149 (131, 168)	130 (112, 148)	289 (213, 365)	114 (70, 158)	82 (48, 115)	117 (79, 155)	92 (53, 131)	326 (255, 397)	77 (40, 114)
2015–2017	162 (143, 181)	143 (124, 162)	289 (215, 364)	130 (84, 176)	109 (70, 148)	97 (62, 132)	98 (58, 138)	386 (307, 465)	89 (50, 129)
2016–2018	168 (148, 188)	149 (129, 169)	294 (220, 369)	124 (79, 169)	136 (91, 181)	107 (69, 145)	103 (61, 144)	404 (321, 488)	72 (36, 108)

^a^ Maternal Newborn Health Registry 2010–2018 deliveries, excluding women lost to follow-up prior to delivery or missing maternal status at 42 days. Clusters collecting data during the enti re period of 2010–2018, or 2014–2018 for DRC

^b^ Maternal Mortality Ratio (MMR) is maternal deaths per 100,000 live births. The 95% confidence interval for the MMR is approximated using the variance of the proportion of maternal deaths for each site and year

## Discussion

Our data show an MMR higher than the SDG 2030 goals in all research sites within the NICHD GN. We measured an MMR of 157 / 100,0000 live births, which is more than double the SDG target of less than 70 per 100,000 live births by 2030. There is great variation in MMR across sites, with the lowest average MMRs during the study period in the Guatemala (97), Kenya (107) and Zambia (107) sites to the highest MMRs measured in the DRC (317) and Pakistan (327) sites. We identified maternal, antenatal and delivery characteristics that were associated with maternal death. We identified hemorrhage, infection and pre-eclampsia/eclampsia as the most common causes of maternal death. Of the 75% of cases where fetal or neonatal outcome were known, more than 20% were associated with stillbirth. We observed some sites with improvement in the MMR over time, but the Pakistani site reported an increase in the MMR over time.

When compared to the World Bank modeled estimates for the MMR by country, our data indicate a lower MMR for the sites in all countries, except Pakistan [17]. For example, we measured an MMR of 317 in the DRC site, compared to the World Bank estimates of 850, and we measured an MMR of 107 in the Zambia site, compared to the World Bank estimate of 398 [7]. The World Bank estimates are developed from representative samples of the populations across countries in contrast to our MMR which measures outcomes in a region within the country. Our data are collected at the individual level within a discrete population within each country. It might not be representative of the entire population of the diverse countries in which we work. For example, our data from the rural provinces in the DRC are likely not reflective of the population in the urban capital of Kinshasa. However, we do not believe that this variation in methodology entirely accounts for the wide discrepancies we have observed. We presume these data highlight the differences between data measured prospectively in a direct fashion, compared to indirect measurements and estimates derived from modelling strategies with unknown reliability.

We describe an increased risk of maternal death among women who deliver in hospitals, those who have deliveries attended by physicians and those who deliver by Caesarean section. We presume that the women who seek care by a physician or in a hospital are at higher risk for pregnancy complications or have experienced pregnancy complications that have required a higher level of medical care. Therefore, we believe that these associations are confounded by indication. This presumption is consistent with previous literature that indicates that the largest proportion of maternal deaths occur in facilities where the higher risk patients are treated and where complicated patients are referred [18]. Ronsmans and colleagues describe three categories of women who die in hospitals: women who arrive in a moribund state too late to benefit from emergency medical treatment, high risk women who could have been saved if they received timely and effective interventions and women who develop serious complications within the hospital [18]. While our data are consistent with findings that hospital delivery is associated with higher maternal mortality, we do not have data on the quality of care that was delivered to these women or at what point in the mother’s illness she arrived at the hospital for care.

The MNHR represents a useful tool for recording and tracking the MMR in several LMICs. Our data are rigorously collected in a prospective fashion with consistent methodology over a long period of time. Because we collect data from all pregnant women living in a geographic area, regardless of delivery site, the MNHR is ideal for giving an accurate account of the MMR in the population studied. The MNHR has a high rate of recruitment and retention (outcomes obtained on 98.6% of women) which provides robust data for studying maternal and neonatal outcomes through 6 post-partum weeks, referenced elsewhere in the supplement. Furthermore, the MNHR contains data from a consistent population of women in LMICs over a 9-year period. This ongoing data collection tool is ideal for examining trends in health outcomes within study regions over time.

The MNHR does have some practical limitations. Women can be enrolled in the MNHR at any point in their pregnancy, so we potentially underrepresent maternal death that occurs early in pregnancy that could be related to miscarriages or MTPs. Our analyses are also limited to the variables that are collected within the MNHR. For example, the causes of maternal death are estimates using interview techniques and medical chart extraction. Supportive laboratory data were rarely available and autopsies were not performed. As such, these data were not included in the cause of death algorithm [16]. Additionally, sites that do not routinely measure blood pressures as part of ANC, report lower rates of pre-eclampsia/eclampsia. However, despite these biases, our approach to identify causes of death found similar relative contributions to other published reports. Therefore, our approach might represent an opportunity to identify maternal health practices that could prevent specific causes of mortality [19, 20].

## Conclusions

The results of our study contribute important MMR data in 6 LMICs. The differences that we described, compared to MMRs from modelling estimates, illustrate the vast variation in MMR estimates given the data source and strategy used. Because our data are collected prospectively, we believe that the MNHR is an ideal source for evaluating key health outcomes. The high, but relatively stable, MMR in many countries highlights an opportunity for improvement in these countries. While the Guatemalan and Zambian sites have demonstrated success in lowering the MMR over the study period, sites in other countries, like the DRC and Pakistan, had persistently high MMRs at the end of the study period. Maternal mortality is an important public health problem and these data confirm the opportunity for improvement.

## Data Availability

The datasets generated and analysed during the current study are not yet publicly available due to ongoing data analyses, but they will be available in the NICHD Data and Specimen Hub. Requests for data prior to the public release will be handled by the authors.

## Abbreviations

SDG: Sustainable Development Goals
MMR: Maternal Mortality Ratio
LMIC: Low and lower-middle income countries
NICHD: National Institute of Health *Eunice Kennedy Shriver National* Institute of Child Health and Human Development
GN: NICHD Global Network for Women’s and Children’s Health Research
MNHR: Maternal Newborn Health Registry
DRC: Democratic Republic of Congo
MTP: Medical Termination of Pregnancy
ANC: Antenatal care
RR: Relative Risk
CI: Confidence Intervals
